# *Sost* deficiency led to a greater cortical bone formation response to mechanical loading and altered gene expression

**DOI:** 10.1038/s41598-017-09653-9

**Published:** 2017-08-25

**Authors:** David Pflanz, Annette I. Birkhold, Laia Albiol, Tobias Thiele, Catherine Julien, Anne Seliger, Erin Thomson, Ina Kramer, Michaela Kneissel, Georg N. Duda, Uwe Kornak, Sara Checa, Bettina M. Willie

**Affiliations:** 1 Julius Wolff Institute, Charité - Universitätsmedizin Berlin, Berlin, Germany; 2 Berlin-Brandenburg School for Regenerative Therapies, Charité - Universitätsmedizin Berlin, Berlin, Germany; 3 Continuum Biomechanics and Mechanobiology Research Group, Institute of Applied Mechanics, University of Stuttgart, Stuttgart, Germany; 4 Novartis Institutes for BioMedical Research, Basel, Switzerland; 5 Institute for Medical Genetics and Human Genetics, Charité- Universitätsmedizin Berlin, Berlin, Germany; 6 Berlin-Brandenburg Center for Regenerative Therapies, Charité-Universitätsmedizin Berlin, Berlin, Germany; 7 Max Planck Institute for Molecular Genetics, Berlin, Germany; 8 Research Centre, Shriners Hospital for Children-Canada, Department of Pediatric Surgery, McGill University, Montreal, Canada

## Abstract

Bone adaptation optimizes mass and structure, but the mechano-response is already reduced at maturation. Downregulation of sclerostin was believed to be a mandatory step in mechano-adaptation, but in young mice it was shown that load-induced formation can occur independent of sclerostin, a product of the *Sost* gene. We hypothesized that the bone formation and resorption response to loading is not affected by *Sost* deficiency, but is age-specific. Our findings indicate that the anabolic response to *in vivo* tibial loading was reduced at maturation in *Sost* Knockout (KO) and littermate control (LC) mice. Age affected all anabolic and catabolic parameters and altered *Sost* and Wnt target gene expression. While load-induced cortical resorption was similar between genotypes, loading-induced gains in mineralizing surface was enhanced in *Sost KO* compared to LC mice. Loading led to a downregulation in expression of the Wnt inhibitor *Dkk1*. Expression of *Dkk1* was greater in both control and loaded limbs of *Sost* KO compared to LC mice suggesting a compensatory role in the absence of *Sost*. These data suggest physical activity could enhance bone mass concurrently with sclerostin-neutralizing antibodies, but treatment strategies should consider the influence of age on ultimate load-induced bone mass gains.

## Introduction

A deficiency in sclerostin, a product of the *Sost* gene, leads to a high bone mass phenotype in humans and mice^1–3^. Thus monoclonal antibodies to block sclerostin (e.g. Romosozumab, Blosozumab)^4, 5^ as treatment options against osteoporosis are currently in Phase III clinical testing for osteoporosis. Since there is little knowledge about the long-term effects of sclerostin inhibition to increase bone mass concurrent treatment strategies are being considered, e.g. physical activity.

It has been postulated that physical activity may not lead to any additional bone gain during sclerostin inhibition, since the down-regulation of sclerostin was thought to be a mandatory step in the anabolic response to loading^6^. However, recently Morse *et al*. showed that young growing 10 week old *Sost KO* mice and strain-matched wild-type C57BL/6 mice had a similar response to loading in terms of cortical thickness (Ct.Th), but a greater increase in MAR and Ps.BFR/BS in response to loading^7^. While these results were observed at the 37% tibial diaphysis, they reported a slightly lower response to loading in Ct.Th for *Sost* KO compared to wild-type mice at the 50% tibial diaphysis region, but a significantly greater response at the metaphyseal region. It is unclear why they observed such a region-specific load-induced cortical bone formation response in the absence of *Sost*. A more recent study by Robling *et al*. showed that load-induced ulnar periosteal bone formation occurred normally in the absence of Sost, although they also observed regional differences in load-induced bone formation in *Sost* KO mice. These regional data suggested that *Sost* is required for localization of new bone to surfaces experiencing high strains. Although these studies address the question of whether the Wnt inhibitor *Sost* is required for the anabolic response to loading in young mice, it remains unclear how Wnt signaling is affected during loading under *Sost* inhibition. Additionally, since both these experiments were performed only in young animals it remains unclear if physical activity will be beneficial in terms of increasing bone mass further in adult individuals, which are more prone to suffer from bone loss.

Experimental studies have shown that the formation response to loading in C57Bl/6 mice is reduced with aging and that this reduced mechano-responsiveness occurs already at skeletal maturation^8–12^. Data from human exercise trials also show that physical activity is more effective in increasing BMD in younger individuals compared to adults or elderly individuals^13, 14^. Sclerostin might play a role in the loss in mechanoresponsiveness with maturation and aging, since it has been shown that sclerostin levels in healthy humans increase with age^15, 16^. However, the role of sclerostin in age-related reduced adaptive (re)modeling (modeling and remodeling) to mechanical loading has never been investigated. In addition to potential anabolic benefits of sclerostin deficiency and mechanical loading, it also remains unclear how resorption is affected by this combined therapy. Several studies have suggested that sclerostin may promote osteoclastic bone resorption through regulation of RANKL^17–22^ and also promote osteocytic osteolysis via stimulation of carbonic anhydrase 2 expression^17^. No study has yet examined the resorptive response to mechanical loading under sclerostin deficiency, only effects of unloading on resorption in *Sost* deficient mice have been studied^7^.

**Figure 1:**
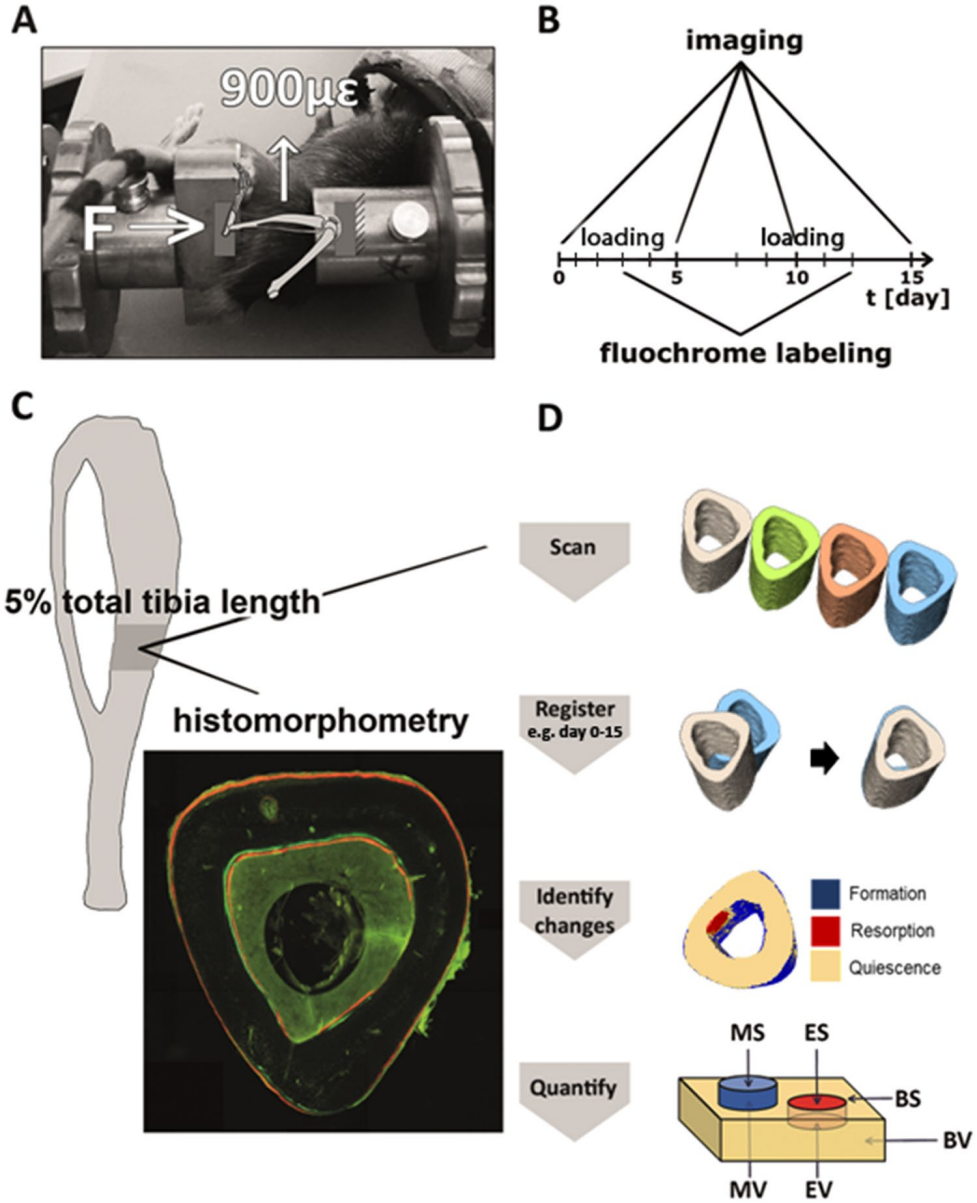
Schematic illustration of the experimental *in vivo* loading setup (**A**), timeline of *in vivo* microCT imaging (day 0, 5, 10, 15) and fluochrome labeling (day 3 and 12) (**B**), histomorphometry and image analysis (**C**). Schematic showing visualization of bone formation (mineralized) and resorption (eroded) volumes and surfaces by quantification of quiescent bone (yellow), newly formed bone (blue), and resorbed bone (red) regions (**D**).

Thus, it remains unclear whether mechanical loading will be effective in enhancing bone formation and reducing bone resorption further in adult patients treated with sclerostin inhibition and whether or how it is affecting Wnt signaling. We hypothesized that the bone formation and resorption response to mechanical loading is not affected by *Sost* deficiency, but is affected by skeletal maturation. We investigated changes in cortical bone morphology and the formation/ resorption response to two weeks of cyclic axial loading of the left tibiae compared to the right non-loaded tibiae in young growing 10 week old and skeletally mature adult 26 week old *Sost* KO mice and littermate control (LC) mice (Fig. 1). We performed *in vivo* microCT imaging at day 0, 5, 10, and 15 and conventional 2D histomorphometry, with labeling at day 3 and 12. *In vivo* time-lapsed 3D morphometry was performed on imaging data at day 0 and 15 to quantify the volume and surface area of bone formation and resorption over the 15 day interval. We also measured gene expression of several Wnt target genes and Wnt inhibitors at 3, 8 and 24 hours after a single loading session in an additional set of mice of both ages and genotypes.

## Results

### Altered load transmission in Sost deficient mice compared to littermate controls

Since we planned to conduct a strain-matched loading study, we first tested the relationship between the applied axial compressive load and the bone tissue deformation engendered at the left tibia. The following load levels were calculated for each group to attain peak strains of +900με at the strain gauge site on the medial surface of the cortical bone mid-diaphyseal: −12.9 N in 10 week old *Sost* KO mice, −14.5 N in 26 week old *Sost* KO mice, −7N in 10 week old LC mice and −7N in 26 week old LC mice. The slopes of the strain-load regressions^23^ were the following: 10wk *Sost* KO mice: (−0.0143 ± 0.0021 N/με), 26wk *Sost* KO mice (−0.0161 ± 0.0016 N/με), 10wk LC mice: (−0.0077 ± 0.0016 N/με), and 26 wk old LC mice (−0.0079 ± 0.0006 N/με). The difference in the applied forces illustrates the strong divergence of the thickness and shape of the cortical bone in LC and *Sost* KO.

### Load-induced increases in periosteal bone formation rates and newly mineralized surface area were significantly greater in Sost KO than LC mice

**Figure 2:**
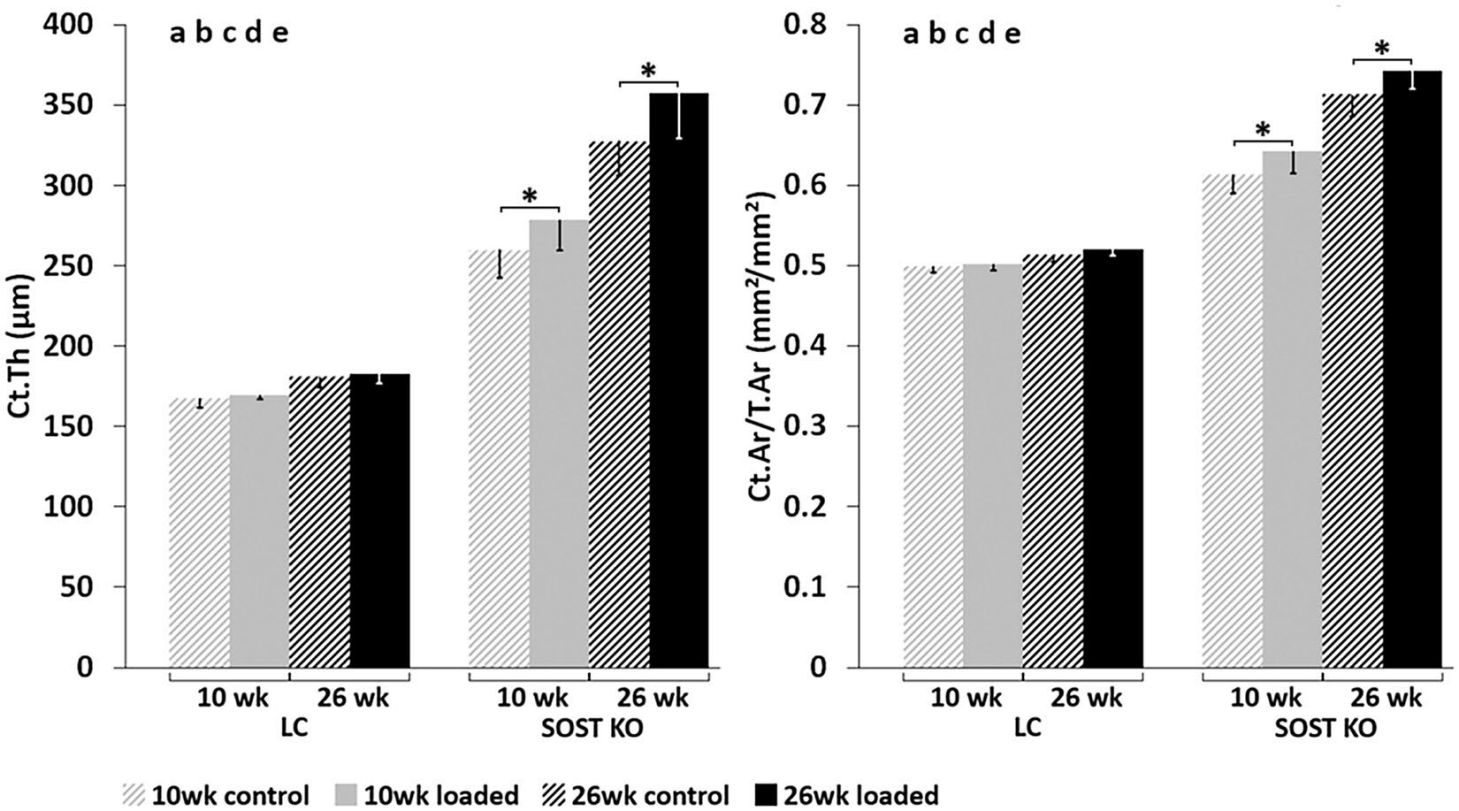
MicroCT results of cortical thickness (Ct.Th) and cortical area normalized to total area (Ct.Ar/T.Ar) at day 15. ANOVA: indicates an effect of (**a**) genotype, (**b**) age, (**c**) loading, (**d**) genotype & age, (**e**) genotype & loading, (**f**) age & loading, p < 0.05. Asterisk indicates a significant difference between loaded and control bones (Paired t-test; p < 0.05).

**Figure 3:**
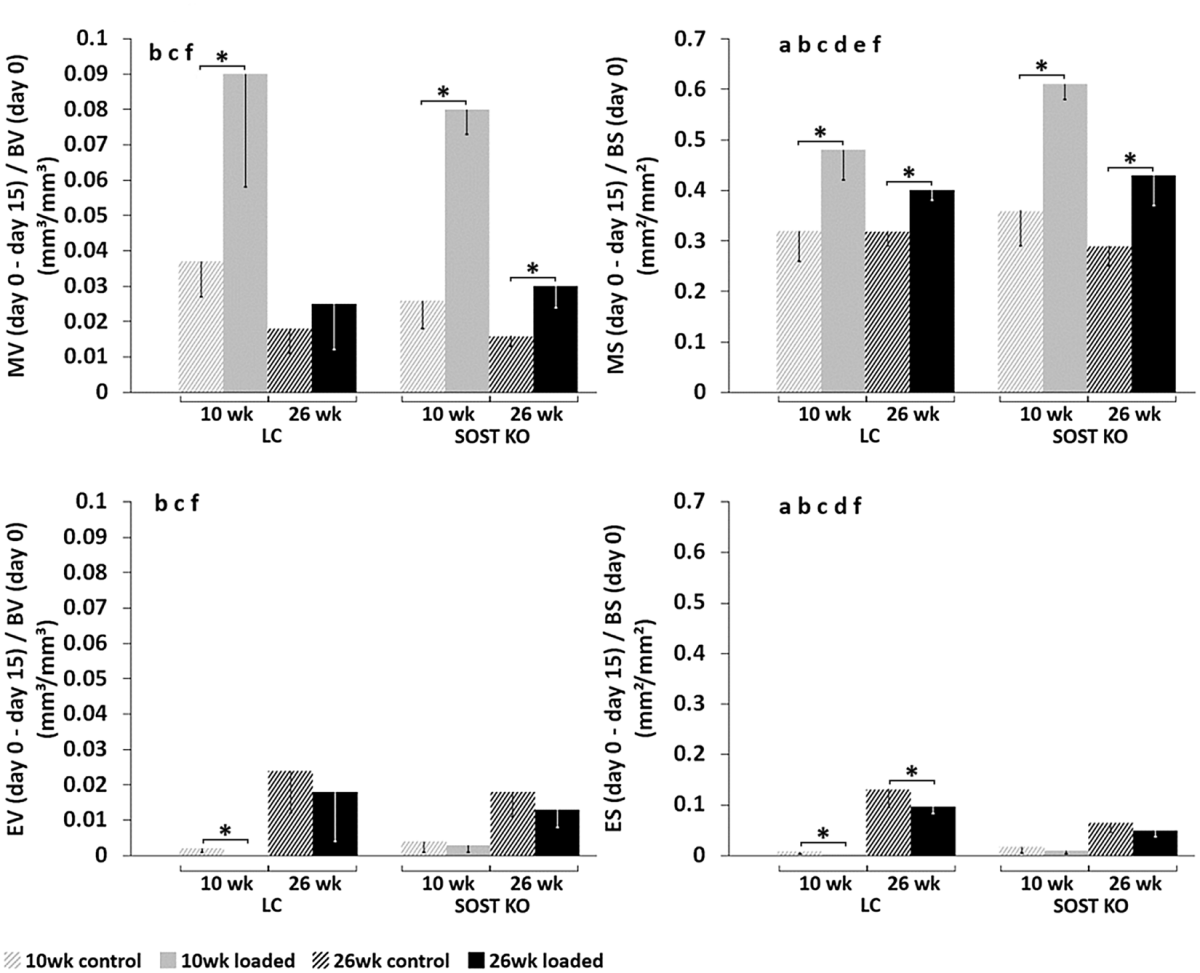
3D dynamic time lapsed *in vivo* morphometry of mineralizing volume (MV) and surface (MS) as well as eroded volume (EV) and surface (ES) at the tibial mid-diaphysis. All parameters are expressed as the amount of newly formed or resorbed bone between day 0 and day 15, normalized to the bone volume (BV) or bone surface (BS) at day 0, which includes both the periosteal and endocortical surfaces. ANOVA: indicates an effect of (**a**) genotype, (**b**) age, (**c**) loading, (**d**) genotype & age, (**e**) genotype & loading, (**f**) age & loading, p < 0.05. Asterisk indicates a significant difference between loaded and control tibiae (Paired t-test; p < 0.05).

**Figure 4:**
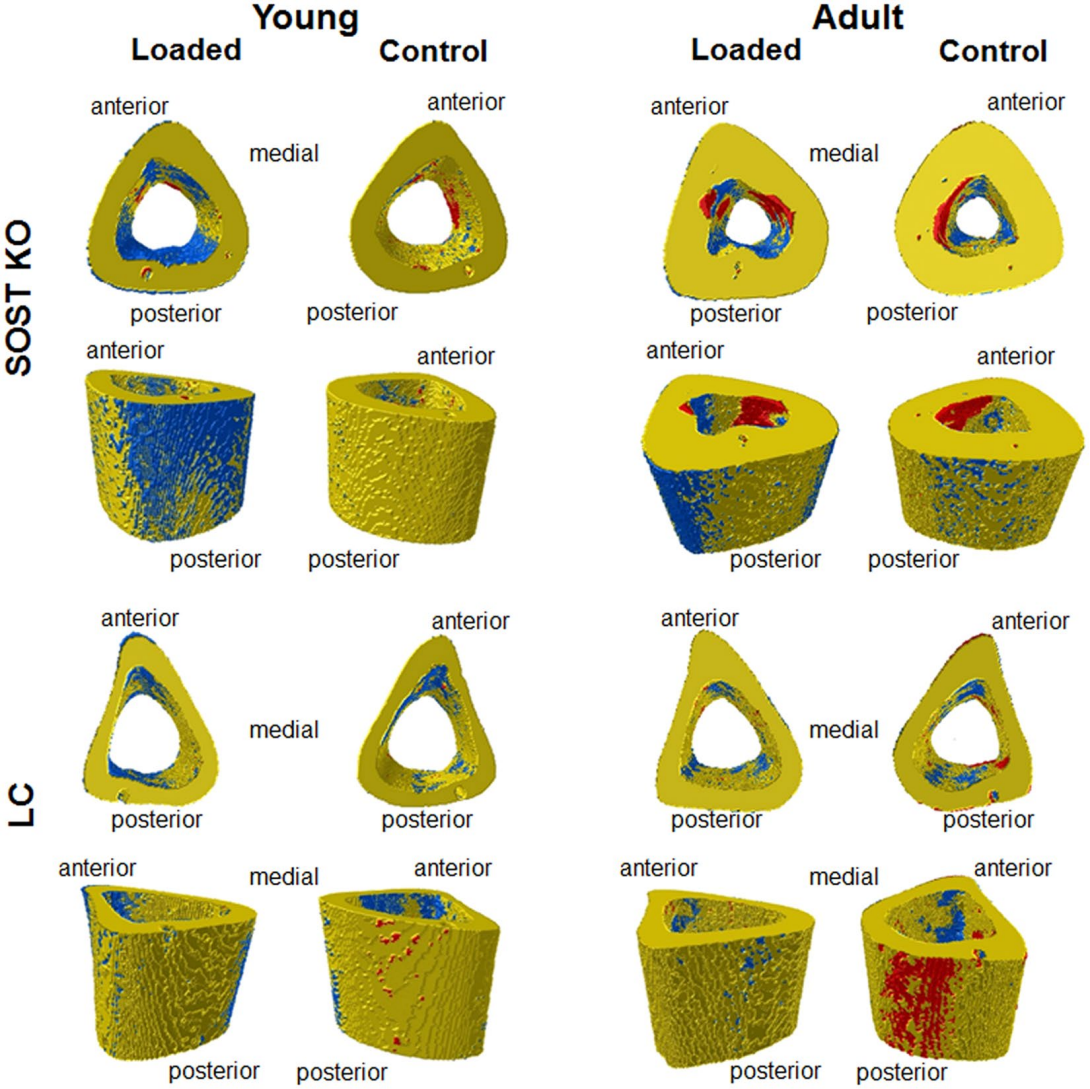
Visualization of (re)modeling occurring over the 15 day experimental period in the left loaded tibia and right control tibia of young and adult *Sost* KO and LC mice. The newly formed (blue), resorbed (red), and quiescent (yellow) bone can be seen on the periosteal and endocortical surfaces.

**Figure 5:**
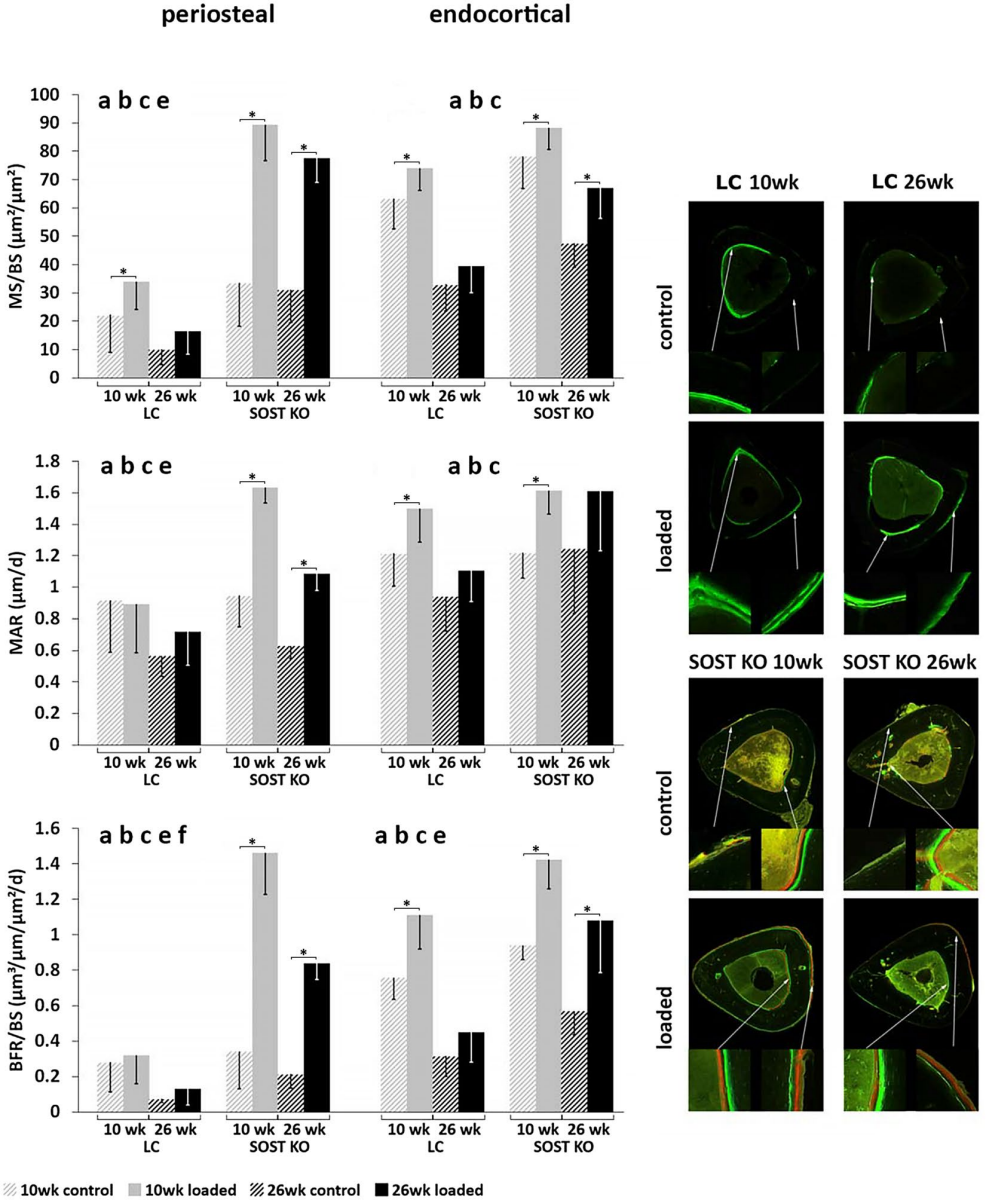
Cortical histomorphometry of mineral apposition rate (MAR) and bone formation rate normalized to bone surface (BFR/BS) after fluorochorme labeling of the bone at day 3 and 12. ANOVA: indicates an effect of (**a**) genotype, (**b**) age, (**c**) loading, (**d**) genotype & age, (**e**) genotype & loading, (**f**) age & loading, p < 0.05. Asterisk indicates a significant difference between loaded and control tibiae (Paired t-test; p < 0.05).

A significant anabolic response to loading was observed in static and dynamic microCT parameters as well as histomophometric measures of bone formation (Figs 2–5). Subanalyses of each genotype showed that at the beginning of the experiment (day 0), there were no difference in static microCT parameters (Ct.Th, Ct.Ar, Ct.Ar/T.Ar) between the left (loaded) and right (control) tibia, (inter-limb difference) for either *Sost* KO mice or LC mice of the same age (10 or 26 weeks old) (Table 1). By day 15, static microCT parameters were greater in the loaded compared to control limb of both young (Ct.Ar: +8, Ct.Ar/T.Ar: +3%, Ct.Th: +7%) and adult (Ct.Ar: +5, Ct.Ar/T.Ar: +4%, Ct.Th: +9%) *Sost* KO mice (p < 0.014, Fig. 2, Table 1). In contrast, there were no significant differences in static microCT parameters measured in the loaded and control limbs of either young or adult LC mice (Fig. 2, Table 1).

**Table 1:** Cortical (Ct) bone parameters of the tibial midshaft, determined by *in vivo* microCT at days 0, 5, 10 and 15 in mice exposed to axial compression of 900 µɛ (left tibia dynamically loaded, right tibia nonloaded control) (mean ± SD); ANOVA for day 15: indicates an effect of (a) genotype, (b) age, (c) loading, (d) genotype & age, (e) genotype & loading, (f) age & loading p < 0.05.

Outcome	LC mice				*Sost* KO mice			
	10 wk old		26 wk old		10 wk old		26 wk old	
	Control	Loaded	Control	Loaded	Control	Loaded	Control	Loaded
Day 0	(n = 7)	(n = 7)	(n = 7)	(n = 7)	(n = 7)	(n = 7)	(n = 7)	(n = 7)
Imax (mm^4^)	0.06 ± 0.01	0.06 ± 0.01	0.07 ± 0	0.07 ± 0.01	0.13 ± 0.02	0.14 ± 0.02	0.22 ± 0.01	0.22 ± 0.01
Imin (mm^4^)	0.04 ± 0.01	0.04 ± 0	0.06 ± 0	0.06 ± 0	0.11 ± 0.01	0.11 ± 0.01	0.18 ± 0.01	0.19 ± 0.01
Ct.Ar (mm²)	0.46 ± 0.02	0.45 ± 0.03	0.57 ± 0.02	0.55 ± 0.03	0.85 ± 0.05	0.85 ± 0.06	1.25 ± 0.05	1.27 ± 0.06
T.Ar (mm²)	0.95 ± 0.05	0.96 ± 0.05	1.05 ± 0.06	1.05 ± 0.04	1.41 ± 0.09	1.42 ± 0.08	1.75 ± 0.03	1.75 ± 0.04
Ct.Ar/T.Ar (mm²/mm²)	0.48 ± 0.01	0.46 ± 0.01	0.54 ± 0.02	0.52 ± 0.01	0.6 ± 0.02	0.6 ± 0.02	0.72 ± 0.03	0.72 ± 0.02
Ct.Th (µm)	158 ± 5	152 ± 6	185 ± 7	184 ± 8	254 ± 13	250 ± 15	337 ± 16	346 ± 19
Ct.vTMD (mg HA/cm^3^)	1263 ± 13	1239 ± 21	1316 ± 19	1320 ± 13	1268 ± 16	1263 ± 19	1309 ± 8	1326 ± 24
Day 5	(n = 7)	(n = 7)	(n = 7)	(n = 7)	(n = 7)	(n = 7)	(n = 4)	(n = 4)
Imax (mm^4^)	0.06 ± 0.01	0.06 ± 0.01	0.07 ± 0	0.07 ± 0.01	0.13 ± 0.01	0.14 ± 0.02	0.21 ± 0.01	0.22 ± 0.02
Imin (mm^4^)	0.04 ± 0.01	0.04 ± 0.01	0.06 ± 0.01	0.06 ± 0	0.11 ± 0.01	0.11 ± 0.01	0.18 ± 0.01	0.19 ± 0.02
Ct.Ar (mm²)	0.47 ± 0.04	0.46 ± 0.03	0.57 ± 0.02	0.55 ± 0.03	0.86 ± 0.06	0.86 ± 0.07	1.24 ± 0.05	1.29 ± 0.08
T.Ar (mm²)	0.98 ± 0.08	0.97 ± 0.05	1.09 ± 0.03	1.06 ± 0.04	1.39 ± 0.06	1.41 ± 0.08	1.72 ± 0.04	1.75 ± 0.08
Ct.Ar/T.Ar (mm²/mm²)	0.48 ± 0.01	0.47 ± 0.01	0.52 ± 0.01	0.52 ± 0.01	0.61 ± 0.03	0.61 ± 0.03	0.72 ± 0.03	0.74 ± 0.01
Ct.Th (µm)	160 ± 7	156 ± 5	184 ± 6	183 ± 7	259 ± 20	255 ± 20	342 ± 23	352 ± 24
Ct.vTMD (mg HA/cm^3^)	1264 ± 20	1234 ± 19	1314 ± 19	1316 ± 7	1270 ± 34	1261 ± 16	1313 ± 8	1363 ± 27
Day 10	(n = 7)	(n = 7)	(n = 7)	(n = 7)	(n = 7)	(n = 7)	(n = 7)	(n = 7)
Imax (mm^4^)	0.06 ± 0.01	0.06 ± 0.01	0.08 ± 0.01	0.07 ± 0.01	0.13 ± 0.02	0.15 ± 0.02	0.21 ± 0.02	0.23 ± 0.03
Imin (mm^4^)	0.04 ± 0.01	0.04 ± 0.01	0.06 ± 0.01	0.06 ± 0.01	0.11 ± 0.01	0.12 ± 0.01	0.18 ± 0.01	0.19 ± 0.02
Ct.Ar (mm²)	0.48 ± 0.03	0.47 ± 0.03	0.57 ± 0.03	0.55 ± 0.03	0.86 ± 0.06	0.91 ± 0.07	1.25 ± 0.06	1.29 ± 0.06
T.Ar (mm²)	0.98 ± 0.07	0.96 ± 0.05	1.09 ± 0.04	1.06 ± 0.04	1.41 ± 0.06	1.46 ± 0.06	1.72 ± 0.07	1.76 ± 0.09
Ct.Ar/T.Ar (mm²/mm²)	0.49 ± 0.01	0.49 ± 0.01	0.52 ± 0.01	0.52 ± 0.01	0.61 ± 0.02	0.62 ± 0.03	0.72 ± 0.02	0.73 ± 0.02
Ct.Th (µm)	164 ± 6	163 ± 5	183 ± 7	184 ± 5	260 ± 17	264 ± 21	340 ± 15	347 ± 18
Ct.vTMD (mg HA/cm^3^)	1283 ± 22	1252 ± 27	1333 ± 35	1318 ± 11	1262 ± 16	1275 ± 25	1315 ± 6	1325 ± 11
Day 15	(n = 6)	(n = 6)	(n = 7)	(n = 7)	(n = 7)	(n = 7)	(n = 7)	(n = 7)
Imax (mm^4^) a, b, c, d, e	0.06 ± 0.01	0.06 ± 0	0.07 ± 0.01	0.07 ± 0.01	0.14 ± 0.02	0.15 ± 0.02	0.22 ± 0.01	0.22 ± 0.02
Imin (mm^4^) a, b, c, d, e	0.04 ± 0.01	0.05 ± 0	0.06 ± 0.01	0.06 ± 0.01	0.11 ± 0.01	0.12 ± 0.01	0.18 ± 0.01	0.19 ± 0.02
Ct.Ar (mm²) a, b, c, d, e	0.49 ± 0.03	0.5 ± 0.02	0.56 ± 0.02	0.55 ± 0.03	0.87 ± 0.06*	0.94 ± 0.06	1.24 ± 0.06*	1.3 ± 0.06
T.Ar (mm²) a, b, c, e	0.97 ± 0.05	0.99 ± 0.04	1.08 ± 0.04*	1.06 ± 0.04	1.42 ± 0.07	1.46 ± 0.05	1.74 ± 0.05	1.75 ± 0.06
Ct.Ar/T.Ar (mm²/mm²) a, b, c, d, e	0.5 ± 0.01	0.5 ± 0.01	0.51 ± 0.01	0.52 ± 0.01	0.61 ± 0.02*	0.64 ± 0.03	0.71 ± 0.03*	0.74 ± 0.02
Ct.Th (µm) a, b, c, d, e	168 ± 6	170 ± 3	181 ± 7	183 ± 6	260 ± 18*	278 ± 19	328 ± 22*	357 ± 28
Ct.vTMD (mg HA/cm^3^) a, b, e	1279 ± 11*	1255 ± 13	1321 ± 14*	1310 ± 9	1255 ± 17*	1273 ± 26	1315 ± 10*	1330 ± 8

Asterisk indicates a significant difference between loaded and control tibiae for each age and genotype (Paired t-test; p < 0.05).

Subanalyses of the dynamic microCT measures of each genotype showed that the loaded limb had a significantly greater volume of newly formed bone than the control limb in young (MV/BV_day0–15_: +208%) and adult (MV/BV_day0–15_ +88%) *Sost* KO mice over the 15 day period (p < 0.02, Fig. 3). Whereas, a significantly increased volume of newly formed bone in the loaded compared to the control limbs was also observed in the young (MV/BV_day0–15_: +143%, p = 0.018), but not in the adult (MV/BV_day0–15_: +39%, p > 0.05) LC mice (Figs 3 and 4).

Histomorphometric bone formation parameters were also significantly increased by loading in young *Sost* KO mice (e.g. Ec.BFR/BS: +51%, Ps.BFR/BS: +329%) and adult *Sost* KO mice: (e.g. Ec.BFR/BS: +89%, Ps.BFR/BS: +300%) (p < 0.026, Fig. 5, Table 2). Several histomorphometric bone formation parameters were also greater in the loaded compared to the control limbs of young (e.g. Ec.MS/BS: +17%, +46%, Ps.MS/BS: +52%) and adult (Ec.sLS/BS: +17%) LC mice (p < 0.048, Fig. 5, Table 2).

**Table 2:** Endocortical (Ec) and periosteal (Ps) bone parameters of the tibial midshaft determined by dynamic histomorphometry, based on fluorochrome injections administered on days 3 and 12.

	LC mice				*Sost* KO mice			
	10 wk old		26 wk old		10 wk old		26 wk old	
	Control	Loaded	Control	Loaded	Control	Loaded	Control	Loaded
Outcome	(n = 7)	(n = 7)	(n = 7)	(n = 7)	(n = 6)	(n = 7)	(n = 7)	(n = 7)
Ec.sLS/BS (%) b, f	20.6 ± 7	11.9 ± 4.6	28.1 ± 7.4*	38.7 ± 10.1	23.6 ± 20.4	7.3 ± 7.6	27.7 ± 15.4	23.5 ± 9.3
Ec.dLS/BS (%) a, b, c, e	53.1 ± 11.3*	68.2 ± 7.7	19 ± 10	20.2 ± 10.8	66.5 ± 19.8*	84.6 ± 9.2	33.8 ± 6.3*	55.3 ± 13.5
Ec.MS/BS (%) a, b, c	63.5 ± 10.9*	74.2 ± 8	33.1 ± 9.3	39.5 ± 9.5	78.3 ± 11.4*	88.2 ± 7.7	47.6 ± 8.5*	67.1 ± 10.8
Ec.MAR (μm/day) a, b, c	1.21 ± 0.21*	1.5 ± 0.21	0.94 ± 0.22	1.11 ± 0.2	1.22 ± 0.16*	1.61 ± 0.15	1.24 ± 0.41	1.61 ± 0.38
Ec.BFR/BS (μm³/μm²/day) a, b, c, e	0.76 ± 0.12*	1.11 ± 0.19	0.32 ± 0.12	0.45 ± 0.17	0.94 ± 0.08*	1.42 ± 0.16	0.57 ± 0.13*	1.08 ± 0.29
Ps.sLS/BS (%) a, e	25.6 ± 12	28.7 ± 14	21.3 ± 4.8	24.9 ± 16.9	49.4 ± 25.6	12.4 ± 13.2	35 ± 8.2	37.1 ± 10.7
Ps.dLS/BS (%) a, b, c, e	13.3 ± 7.5	19.6 ± 13.2	1.9 ± 1.6*	4.8 ± 3	13.4 ± 7.5*	83.2 ± 19.4	16 ± 10.2*	59.1 ± 13.8
Ps.MS/BS (%) a, b, c, e	22.4 ± 13.2*	34 ± 9.9	11.9 ± 3.6	16.5 ± 8.2	33.6 ± 15.4*	89.4 ± 12.8	31.2 ± 11.5*	77.7 ± 8.7
Ps.MAR (μm/day) a, b, c, e	0.92 ± 0.33	0.89 ± 0.31	0.57 ± 0.13	0.72 ± 0.22	0.95 ± 0.19*	1.63 ± 0.1	0.63 ± 0.08*	1.09 ± 0.11
Ps.BFR/BS (μm³/μm²/day) a, b,c, e, f	0.28 ± 0.17	0.32 ± 0.16	0.07 ± 0.02	0.13 ± 0.09	0.34 ± 0.21*	1.46 ± 0.23	0.21 ± 0.08*	0.84 ± 0.09

Mice were exposed to axial compression of 900µɛ (left tibia dynamically loaded, right tibia nonloaded control) (mean ± SD); ANOVA: indicates an effect of (a) genotype, (b) age, (c) loading, (d) genotype & age, (e) genotype & loading, (f) age & loading, p < 0.05. Asterisk indicates a significant difference between loaded and control tibiae for each age and genotype (Paired t-test; p < 0.05).

Surprisingly, the response to loading in terms of newly mineralized surface area (MS/BS_day0–15_) and nearly all static microCT and histomorphometric parameters was significantly greater in *Sost* KO mice compared to LC mice (Figs. 3–5 and Tables 1, 2; effect of (e) genotype & loading p < 0.05). Whereas the formation response to loading in terms of volume of newly mineralized bone (MV/BV_day0–15_) was not significantly different between the *Sost* KO and LC mice (Fig. 3, effect of (e) genotype & loading p = 0.625). A subanalysis examining the interlimb difference showed that the response to loading in young Sost KO animals was significantly greater than in LC mice, including the relative MS/BS_day0–15_, nearly all relative day 15 static microCT parameters (rCt.Ar, rCt.Ar/T.Ar, rCt.Th, rCt.vTMD), all relative periosteal histomorphometric parameters, but none of the relative endocortical bone formation indices. The same analysis in the adult mice showed the *Sost* KO mice had a significantly greater response to loading measured in all relative day 15 static microCT parameters (except rT.Ar) and most relative histomorphometric parameters (rEc.dLS/BS, rEc.BFR/BS, Ps.dLS/BS, rPs.MS/BS, rPs.MARand rPs.BFR/BS). Within both, young and adult mice, *Sost* KO mice had a greater load-induced total bone gain (Ct.Ar/T.Ar, Ct.Th) than LC mice (Fig. 2, Table 1). In adult mice this increased cortical area and thickness was achieved through a higher load-induced bone formation at both the periosteal and endocortical surface of *Sost* KO compared to LC mice (p < 0.001, Fig. 5). In young mice, the endocortical surface of both genotypes showed a similar load-induced increase in histomorphometric parameters, the periosteal surface showed a higher load-induced response to loading in young *Sost* KO compared to young LC mice (p < 0.001, Fig. 5).

**Figure 6:**
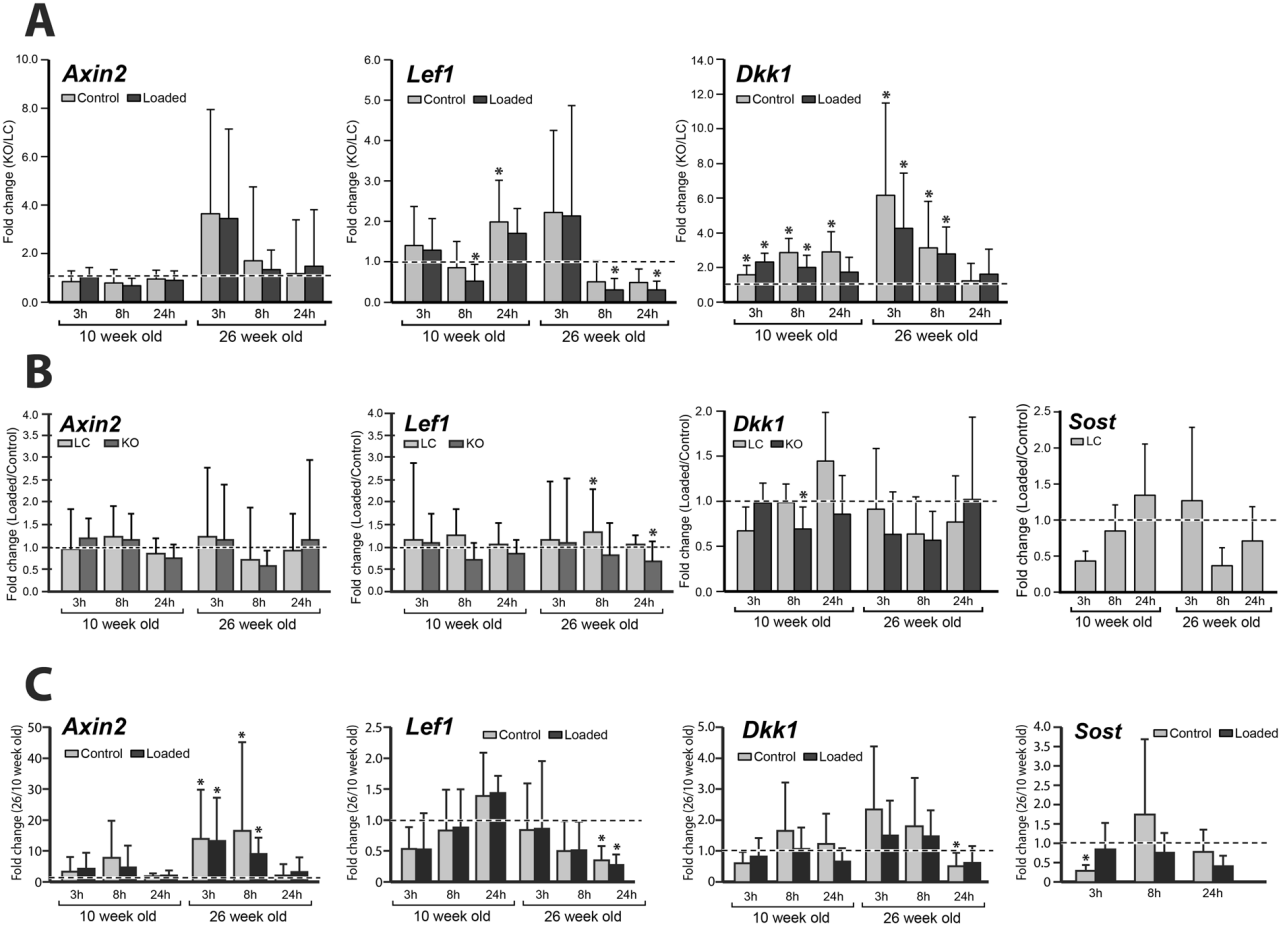
Gene expression of *Axin2, Lef1* and *DKK1* and *Sost* was measured at 3, 8, and 24 hours after a single loading session in the left loaded and right nonloaded tibia of 10 and 26 week old female *Sost* KO and LC mice. Note: *Sost* expression was only measured in LC mice. (**A**) Fold changes in gene expression for *Sost* KO normalized to LC mice. Asterisk indicates a significant difference between *Sost* KO compared to LC for each condition (t-test; p < 0.05). (**B**) Fold changes in gene expression of loaded limb normalized to control limbs are shown. Asterisk indicates a significant difference between loaded and control bones for each condition (paired t-test; p < 0.05). (**C**) Fold changes in gene expression of 26 week old mice normalized to 10 week old mice are shown. Asterisk indicates a significant difference between 26 and 10 week old mice for each condition (t-test; p < 0.05).

The loaded limb had significantly decreased expression of the Wnt inhibitor *Dkk1* compared to the nonloaded control limb of young *Sost* KO mice at 8 hrs. We also observed a tendency for lower *Sost* expression in the loaded limb compared to the nonloaded control limb of LC mice at 3 and 8 hours in 10 week old mice and at 8 and 24 hours in 26 weeks old (Fig. 6B). We found that loading had no effect on expression of the Wnt target gene *Axin2*, while the Wnt target gene *Lef1* had significantly increased expression in loaded compared to control limbs of LC mice at 8 hrs. Loading led to significantly decreased expression of *Lef1* in *Sost* KO mice at 24 hrs (Fig. 6B).

Comparisons of the loaded limbs from *Sost* KO versus LC using independent t-tests showed that the Wnt inhibitor *Dkk1* was significantly upregulated in loaded limbs of *Sost* KO compared to LC mice (Fig. 6A). Also in the loaded limbs, there was a significantly lower expression of *Lef1* in young *Sost* KO mice at 8 hrs and adult *Sost* KO mice at 8 hr and 24 hrs compared to LC mice (Fig. 6A).

### The cortical bone resorption response to loading is similar between Sost KO and LC mice

Since young *Sost* KO and LC mice had minimal resorption over the 15 day period, as observed in the control limb, a reduction in resorption with loading was hardly possible (Fig. 3). Although a significant effect of loading was observed in both EV/BV_day0–15_ and ES/BS_day0–15_ (Fig. 3), sub-analyses of only adult mice showed that for both genotypes, there was a non-significant reduction in the volume of resorbed bone (EV/BV_day0–15_), but a significant reduction in the resorbed surface area (ES/BS_day0–15_) over the 15 day period in loaded compared to control limbs (p≤ 0.05). The percent difference in the volume and surface area of resorbed bone for the loaded compared to control limbs was similar for the adult *Sost* KO mice (EV/BV_day0–15_: −28%, ES/BS_day0–15_: −25%) and LC mice (EV/BV_day0–15_: −25%, ES/BS_day0–15_: −27%). Genotype did not significantly influence the EV/BV_day0–15_, although *Sost* KO mice had a significantly lower ES/BS_day0–15_ than LC mice (Fig. 3). The resorption response to loading (EV/BV_day0–15_ and ES/BS_day0–15_) was not significantly different between the *Sost* KO and LC mice (Fig. 3, effect of (e) genotype & loading p > 0.358). A subanalysis examining the interlimb difference showed that within both ages, the relative EV/BV_day0–15_ and relative ES/BS_day0–15_ were similar between the *Sost* KO and LC mice.

### Skeletal maturation diminished the bone formation response to loading independent of sclerostin

Both genotypes were less mechanoresponsive (in terms of volume and area of newly mineralized bone in response to loading) in adult compared to young mice (Fig. 3, effect of (f) age & loading p < 0.002). For example, the load-induced percent increase in MV/BV_day0–15_ was greater in young (+143%) compared to adult (+39%) LC as well as for young (+207%) compared to adult (+87%) *Sost*KO mice (Fig. 3). A subanalysis examining the interlimb difference showed that within both genotypes, the relative MV/BV_day0–15_ and relative MS/BS_day0–15_ were significantly greater in young compared to adult. In *Sost* KO mice, maturation diminished the bone formation response to loading in the periosteal cortical bone (Ps.BFR/BS: −34%, Ps.MAR: −35%) (p < 0.046, Fig. 5) and therefore the overall bone formation response (MV/BV_day0–15_: −75% and MS/BS_day0–15_: −46%) (p < 0.011, Fig. 3). Interestingly there was no difference in mechanoresponse to loading at the endocortical surface between young and adult *Sost* KO mice; relative (interlimb) endocortical bone formation histomorphometric indices were similar. Whereas the relative Ec.BFR/BS was (−62%) significantly lower in adult compared to young LC mice (Fig. 5). Age significantly affected *Sost* expression in LC mice as well as Wnt target genes *Lef1* and *Axin2* expression in *Sost* KO and LC mice (Fig. 6C). Interestingly, the expression of *Lef1* at the 24 hr time point was significantly lower in both the loaded and control limbs of adult compared to young *Sost* KO mice. At the 3 and 8 hr time points, *Axin2* expression was significantly greater in both the loaded and control limbs of adult compared to young *Sost* KO mice. We also observed a significantly greater *Dkk1* expression at 24 hrs in the control limbs of young compared to adult *Sost* KO mice. Lastly, we measured a significantly lower *Sost* expression at 3 hrs in the control limb of adult compared to young LC mice.

### Sost deficiency and maturation altered bone formation, resorption and gene expression in the nonloaded control tibia

The control tibia of adult LC mice was larger than the control tibia of young LC mice according to microCT parameters: Ct.Ar: +14%, T.Ar: +11%, Ct.Th: +8% (p < 0.017, Fig. 2, Table 1). However, dynamic histomorphometry and 3D morphometry data showed that less newly mineralized bone was formed (e.g. Ec.MS/BS: −48%, Ps.MS/BS: −47%, MV/BV_day0–15_: −19%, p < 0.044) and more bone was resorbed (ES/BS_day0–15_: +11.5%, p = 0.002 and EV/BV_day0–15_: +2.2%, p = 0.001) in control limbs of adult compared to young LC mice over the 15 day experimental period (Figs. 3–5).

Control limbs from adult *Sost* KO mice also had significantly greater cortical bone parameters than young *Sost* KO mice (Ct.Ar: +43%, T.Ar: +23%, Ct.Ar/T.Ar: +16%, Ct.Th: +26%) (p = 0.001, Fig. 2, Table 1). Similar to the LC mice, bone resorption indices were higher (EV/BV_day0–15_: +1.4%, ES/BS_day0–15_: +4.7%, p = 0.001) and formation indices lower (e.g. MV/BV_day0–15_: −1%, p < 0.02) in control limbs from adult compared to young *Sost* KO mice (Figs 3–5).

Additionally, the difference in cortical bone parameters between the control limbs of young and adult mice were much greater in *Sost* KO compared to LC mice (e.g. Ct.Ar: +428%, p = 0.001, Ct.Th: +423%, p = 0.001) (Table 1). Young *Sost* KO mice had higher endocortical bone formation than LC mice of the same age in the control limb (Ec.MS/BS: +23%, Ec.BFR/BS: +24%) (p < 0.035, Fig. 5). Adult *Sost* KO control limbs showed higher endocortical and even more pronounced periosteal bone formation compared to LC control limbs of the same age (Ec.MS/BS: +44%, Ec.BFR/BS: +80%, Ps.MS/BS: +162%, Ps.BFR/BS: +190%) (p < 0.0103, Fig. 5, Table 2).

Control limbs of young and adult LC mice had a similar volume of resorption to that of age-matched *Sost* KO mice (EV/ BV_day0–15_ (mm²/mm²) LC 10wk: 0.002 ± 0.001; *Sost* KO 10wk: 0.004 ± 0.003; LC 26wk 0.024 ± 0.012; *Sost* KO 26wk 0.018 ± 0.007). However, adult LC mice had a significantly greater bone resorption surface than adult *Sost* KO mice (ES/BS_day0–15_: +103%, p = 0.001), while young mice of both genotypes exhibited near negligible levels. Therefore in the control limbs, the difference in bone resorption surface between young and adult mice was much greater in LC compared to *Sost* KO mice.

Comparisons of the control limbs from *Sost* KO versus LC showed that the Wnt inhibitor *Dkk1* was significantly upregulated in control limbs of *Sost* KO compared to LC mice (Fig. 6A). We also measured significantly greater expression of *Lef1* at 24 hrs in the control limb of young *Sost* KO compared to LC mice.

## Discussion

In this study, we examined whether two weeks of *in vivo* tibial loading could enhance bone formation and reduce bone resorption in young and adult female Sost KO and LC mice. We hypothesized that the bone formation and resorption response to mechanical loading is not affected by *Sost* deficiency, but is affected by skeletal maturation. We investigated changes in cortical bone morphology and the adaptive (re)modeling response using *in vivo* microCT imaging, registered microCT data as a 4D imaging biomarker of bone formation and resorption, and conventional 2D histomorphometry. We also examined the gene expression in Wnt target genes (*Axin2* and *Lef1*) and Wnt inhibitors (*Dkk1* and *Sost*) at 3, 8, and 24 hours after a single loading session. We observed that *Sost* deficiency not only led to increased formation, but also decreased resorption in the control nonloaded limbs. Our results show that the bone (re)modeling response and the expression of Wnt target genes is dependent on skeletal maturation in *Sost* KO mice (Fig. 6C). *Sost* KO mice had significantly higher *Dkk1* expression compared to LC mice, independent of loading at nearly all time points and ages. We demonstrated that the cortical bone resorption response to loading was similar in *Sost* KO and LC mice, while the cortical adaptive response in terms of formation was enhanced in *Sost* KO compared to LC mice. Additionally, recent finite element analyses from our group showed that compressive and tensile strains were lower in the *Sost* KO than in the LC mice, for both young and adult animals^24^. Therefore, the greater cortical bone formation response to loading in *Sost* KO mice compared to LC mice was not due to higher mechanical strains engendered during loading.

The cortical adaptive response in terms of formation was surprisingly enhanced in *Sost* KO compared to LC mice (Figs 2–5). Although the formation response to loading in terms of volume of newly mineralized bone (MV/BV_day0–15)_ was not significantly different between the *Sost* KO and LC mice, *Sost* KO mice had significantly greater load-induced increases in the absolute and relative newly mineralized surface area (MS/BS_day0–15_), most absolute and relative static microCT parameters and histomorphometric parameters compared to LC mice (Figs 2–5 and Tables 1, 2; effect of (e) genotype & loading p < 0.05 and t-tests of interlimb difference between *Sost* KO and LC mice within each age p < 0.05). A recent study by Robling *et al*.^25^ reported similar load-induced relative periosteal MAR, BFR/BS, and MS/BS in the ulnae between 16 week old female *Sost* KO and wild-type control mice. However, regional analyses showed differences in terms of strain-dependent distribution of newly formed bone in response to loading. They measured greater rBFR/BS in regions of high strain (medial and lateral region) compared to low strain (caudal and cranial region) in wild-type mice. Whereas, *Sost* KO mice had lower rBFR/BS relative to wild-type mice in high strain regions, but greater rBFR/BS relative to wild-type mice in low strain regions. The only other study to examine mechanoresponse during *Sost* deficiency^7^, showed region-specific varied responses to tibial loading in 10 wk old female *Sost* KO mice compared to wild-type controls. In their strain-matched results, they reported a greater response to loading in *Sost* KO mice compared to wild-type at the metaphyseal region (Ct.Th: 23% in Sost KO, 8% in wild-type), but a similar response to loading in *Sost* KO mice compared to wild-type at the 37% mid-diaphyseal region (Ct.Th: 15% in *Sost* KO, 15% in wild-type). At the same region as in our study, 50% mid-diaphyseal region, Morse *et al*. reported a slightly lower response to loading in *Sost* KO compared to wild-type mice (Ct.Th: 11% in Sost KO, 14% in wild-type).We recently showed that the mouse tibial metaphyseal and mid-diaphyseal regions have different set points (or threshold above which an anabolic response to loading occurs) and slopes of the relationship between engendered strains and remodeling response^26^, which may explain the region-specific differences. In addition, some possible reasons for the contrasting results in these studies^7, 25^ compared to our data may be due to differences in loading parameters, anatomical region loaded, or use of wild-types as controls rather than littermates. Also, neither study^7, 25^ included baseline controls which would confirm if the contralateral limb was a suitable control. We included baseline controls in the form of *in vivo* microCT measurements at day 0, prior to loading, where we observed similar interlimb differences between Sost KO and LC mice. Also, Morse *et al*. reported a woven bone response due to loading, which was absent in our study; only lamellar bone was formed in response to loading. There were also stark differences in the reported strain-load relationship of the *Sost* KO mice between the two studies. Morse *et al*.^7^ reported engendering 1200 µε in 10 week old *Sost* KO mice by using −12.5 N, while we engendered 900µɛ in the tibial mid-shaft of young mice using −7N (LC) and −12.9N (*Sost* KO). Originally we loaded a group of 10 week old *Sost* KO mice at a load level (−17N), which through *in vivo* strain gauging we determined engendered 1200µɛ at the mid-diaphysis. However, we had to discontinue the experiment prematurely since this load/strain level led to ankle swelling and limping in the mice (see Method’s section). The difference in the load-strain relationship between the two studies remains unclear, since the *Sost* KO mice in both studies were from the same source. As expected, since the formation response to loading is dependent on the maximum strain levels used^27^, we measured a lower bone formation response to loading with 900  µε in the LC mice in the current study compared to what we reported previously using 1200με in C57Bl/6 wild-type mice^28^. Lastly, previous studies have suggested that load-induced bone formation is inversely proportional to sclerostin abundance^29, 30^, which may have contributed to the different results between the studies.

Our study is the first to examine the effect of mechanical loading on Wnt signaling under sclerostin ablation. Although, Lin *et al*.^31^ showed that unloading via tail suspension led to upregulation of *Sost* and downregulation of *Lef1* expression in 7 and 17 week old female wild-type mice. They measured no alteration in *Lef1* in *Sost* KO mice of either age with unloading. We observed significantly decreased expression of *Lef1* (loaded vs control limb) in *Sost* KO mice at 24 hrs (Fig. 6B). Similar to the significant increased expression of *Lef1* after ulnar loading in young wild-type mice reported by Tu *et al*.^6^, tibial loading in our study led to significantly increased expression (loaded vs control limb) in LC mice at 8 hrs. Our data are consistent with Holguin *et al*.^32^ who reported that loading led to a significantly decreased expression of the Wnt inhibitor *Dkk1* in wild-type mice of various ages. We measured no changes in the expression of the Wnt target gene *Axin2* with loading, which is in line with data of Holguin *et al*.^32^ who report that loading had no effect on expression of Wnt target gene *Axin2* after a single loading bout in wild-type mice. In contrast, they also reported no effect of loading on *Lef1* expression, which we observed in adult LC mice at 8 hrs (Fig. 6B). We could not detect expression of Wnt ligands (*Wnt1* and *Wnt7b*).

Our data also showed that ablation of the *Sost* gene in mice resulted in an increased expression of another Wnt inhibitor, *Dkk1*, possibly due to a feedback mechanism intended to compensate for the loss of *Sost*. However, the bone anabolic response was even higher in *Sost* KO mice, suggesting that *Dkk1* is not sufficient to compensate for the loss of *Sost*. A significantly lower expression of *Lef1* was measured in the loaded limbs of young *Sost* KO mice at 8 hrs and adult *Sost* KO mice at 8 hr and 24 hrs compared to LC mice (Fig. 6A). We also measured significantly greater expression of *Lef1* at 24 hrs in the control limb of young *Sost* KO compared to LC mice. Lin *et al*.^31^ also observed this interaction between genotype and age, as they also reported that young *Sost* KO mice had greater *Lef1* expression than wild-type mice, while there was no difference in *Lef1* expression in adult *Sost* KO compared to wild-type mice.

In both *Sost* KO and LC mice, skeletal maturation led to increased cortical thickness, cortical area fraction and reduced bone formation in the control bones (Figs 2–5 and Tables 1, 2). The increase in bone elastic modulus^33^ and cross-sectional moment of inertia with skeletal maturation would result in a decreased strain in the older mice for a given force, which we did not observe at the medial midshaft. This apparent contradiction can be explained by changes in whole bone geometry with aging. We and others have previously shown that the bones of 26 wk old mice are more curved than those of 10 wk old C57Bl/6 mice^11, 23, 28^, which will increase their bending stresses under a given load and counteract age-related increases in mineral and geometric properties. Additionally, a recent study from our group^24^ showed that although finite element models predicted approximately 900 µε at the strain gauge position, lower strains are predicted in 26 week old mice compared to 10 wk old LC mice at other positions along the tibial length, suggesting that strain gauging one position is inadequate to characterize the strain distribution. The increase in absolute and relative cortical area fraction and cortical thickness due to skeletal maturation in the control bones was higher in *Sost* KO compared to LC mice (Fig. 2). However in both LC and *Sost* KO mice, there was a lower anabolic response to loading in adult mice compared to young mice. The load-induced percent difference in MV/BV_day0–15_ was greater in young (+143%) compared to adult (+39%) LC as well as for young (+207%) compared to adult (+87%) *Sost* KO mice. An age-specific gene expression response was present in both Wnt signaling target genes examined (*Lef1* and *Axin2)*, as well as in *Sost* expression in the LC mice. *Axin2* expression was higher in skeletally mature animals, while *Lef1* was higher in young mice, thereby indicating that different Wnt effectors might be involved in bone metabolism, depending on the skeletal development stage.

We showed that with *Sost* deficiency the load-induced cortical resorption response, in terms of volumes and surface area of resorbed bone in response to loading, was similar to that of LC mice (Fig. 4). As in our previous studies of female C57Bl/6 mice^16, 32^, we observed only a minimal amount of bone resorption occurring in the cortical diaphysis of the tibia from young LC and *Sost* KO mice. There was, however, a small but significant decrease in the eroded surface of the loaded compared to control tibiae of adult LC mice. Similarly, Morse *et al*.^7^ showed no changes with unloading in osteoclast number, osteoclast surface and the fraction of bone surface with osteoclasts, in both young C57BL/6 and *Sost* KO mice. Also, Tian *et al*.^34^ reported that resorption (Ec.Er.S/BS) was similarly reduced at the tibial midshaft of middle-aged rats (10 months old) after 4wks of sclerostin neutralizing antibody treatment in control and unloaded (immobilized) bones. Interestingly, there was a greater eroded surface in the control limbs of adult LC than *Sost* KO mice (Fig. 3). Similarly, it has been shown that short-term sclerostin inhibition leads to reduced bone resorption in ovariectomized rats and cynomolgus monkey vertebrae and femoral endocortex^19, 35, 36^. Moreover, recent studies by Atkins and colleagues show that sclerostin promotes osteoclastic bone resorption through regulation of RANKL and also promotes osteocytic osteolysis via stimulation of carbonic anhydrase 2 expression in human primary osteocyte-like cells and mouse MLO-Y4 cells^17, 22^. Our data suggests that long-term sclerostin deficiency may have a protective influence against age-related increases in cortical bone resorption.

A limitation in our study is that we did not investigate the mechanoresponsiveness of elderly *Sost* KO mice. There were a few reasons for this. Firstly, we learned from 10 and 26wk old *Sost* KO mice that the load levels necessary to achieve comparable strains in elderly *Sost* KO mice would have caused damage to the joints. Secondly, we observed an increased mortality in *Sost* KO mice in our breeding program and therefore, aging a population of *Sost* KO mice would have required an unjustifiable number of mice. Previous studies in wild-type mice from our group and others have shown that most of the reduced formation response to loading occurs already at skeletal maturation and therefore a great deal of information can be gained from examining young and skeletally mature mice^37, 38^. Lastly, in order to reliably mimic the effects of anti-sclerostin treatment it would be necessary to conditionally knock out the *Sost* gene after the growth phase, since treatment is targeting the elderly population with normal bone mass accrual during growth.

Our model aims to investigate structural adaptation of the mouse tibia through surface modeling and remodeling processes to additional mechanical loading. Since these processes include both formation and resorption, we analyze both to understand the structural response of the bone to loading. Models of disuse may be more appropriate for other investigations focused more on resorptive processes. One must keep in mind that the mechanisms responsible for bone formation to additional loading and maintaining bone mass with normal load, may not be the same as those for recovering bone mass after unloading^39^.

Our key findings in the current study are: 1) the load-induced cortical bone formation response was significantly enhanced in *Sost* KO compared to LC mice 2) *Dkk1* expression was significantly greater in *Sost* KO compared to LC mice, and was significantly downregulated with loading (loaded versus control nonloaded limb) 3) the load-induced cortical resorption response was similar in *Sost* KO and LC mice, 4), the anabolic response to loading was reduced at maturation in both LC and *Sost* KO mice coincident with age-dependent expression of Wnt target genes in *Sost* KO and LC mice as well as *Sost* gene expression in LC mice. Although we were not able to obtain data from elderly *Sost* KO mice as explained above, our findings suggest that future treatment strategies which include long-term sclerostin inhibition should consider the age-dependent effectiveness in reducing resorption or increasing formation as well as the role of additional physical activity regimens. Continued understanding of how long-term *Sost/*sclerostin deficiency influences (re)modeling processes is critical to effectively treat patients with low bone mass.

## Materials and Methods

### Animals and genotyping

The sperm of four male *Sost*−/− mice (provided by Novartis) was pooled into two groups and intracytoplasmic sperm injection with the oocytes from female C57Bl/6 mice was performed at the Charité medical university animal facility. The first heterozygous generation was mated among themselves. In following generations, homozygous *Sost* KO and littermate control (LC) mice were identified using a Multiplex PCR with mice tail cuts, according to a protocol provided by Novartis. Three primers (*Sost*-specific endogen: 5′ TCC ACA ACC AGT CGG AGC TCA AGG 3′, *Sost*-specific endogen and target: 5′ ACT CCA CAC GGT CTG GAA AGT TTG G 3′ and Neo target: 5′ GGG TGG GAT TAG ATA AAT GCC TGC TCT 3′, acquired from TIB MOLBIOL, Berlin, Germany) were used to detect the homozygous LC (*Sost*-specific endogen – *Sost* -specific endogen and target) and *Sost* KO (Neo target – *Sost* -specific endogen and target) mice. All animal experiments described were carried out according to the policies and procedures approved by the local legal research animal welfare representative (LaGeSo Berlin, G0021/11).

### *In vivo* strain-load calibration

Single element strain gauges (EA-06-015LA-120, Micromeasurements, USA) were prepared and attached to the medial surface of the tibial mid-shaft aligned with the bone’s long axis of both limbs of 28 mice (n = 7/age/genotype) as explained previously^28, 40^. A range of axial dynamic compressive loads (peak loads ranging from −2 to −14 N in LC and −2 to −20 N in *Sost* KO mice) was applied between the flexed knee and ankle using an *in vivo* loading device (Testbench Electro Force LM1, TA Instruments, USA), while strain measurements were recorded simultaneously using WinTest software to determine the relationship between the applied compressive loads and bone tissue deformation.

### *In vivo* mechanical loading

The left tibiae of 10 week (young) and 26 (adult) week old *Sost* KO and LC mice (n = 7/age/genotype) underwent 2 weeks (5 days/week, Monday-Friday) *in vivo* cyclic compressive loading (216 cycles applied daily at 4 Hz, peak strains at strain-gauge position of +900 με) (Fig. 1) while under anesthesia. The triangle waveform included 0.15 sec symmetric active loading/unloading, with a constant strain rate of 0.016 ε/sec maintained during both the loading and unloading ramp of the waveform in mice of both ages. The waveform also included a 0.1 sec rest phase (−1N) between load cycles and a 5 sec rest inserted between every four cycles. The right tibia served as internal control as described before^28, 41^.

Three days after the last loading session, the mice were sacrificed (day 15), while under anesthesia (ketamine 60 mg/kg and medetomidine 0.3 mg/kg) through an overdose of potassium chloride. The weight was measured before loading and daily throughout the experiment. No mice had to be excluded from the experiment because of weight loss. There were higher weight variations in the *Sost* KO mice (young: 21 g ± 1.5 g; adult: 25.6 g ± 1.5 g) than in the LC mice (young: 19 g ± 0.8 g; adult: 22.9 g ± 0.4 g) at day 15. Additionally, no mice exhibited any complications or signs of limping throughout the experiment.

### Longitudinal *In vivo* micro-computed tomography of cortical mid-diaphysis

Longitudinal *in vivo* micro-computed tomography (microCT) with a voxel size of 10.5μm (vivaCT 40, Scanco Medical, Brüttisellen, Switzerland; 55kVp source voltage, 145 μA source current, 300 ms integration time, no frame averaging, range of 180 degrees) was performed at day 0, 5, 10, and 15 to assess the cortical bone compartment in both the right and left tibiae. Average scan time was 48 minutes. For each imaging session, mice were anesthetized (ketamine 60 mg/kg and medetomidine 0.3 mg/kg). To prevent motion artifacts during microCT scanning, anaesthetized mice were constrained in a custom-made plastic mouse bed. Scans were reconstructed and analyzed using standard filtered backprojection implemented using software from the microCT. The microCT was calibrated weekly against a hydroxyapatite mineral phantom for determining in-plane spatial resolution. An error occurred during imaging of one young LC mouse taken at day 15, and thus had to be excluded; otherwise microCT data was obtained at all-time points for the right and left tibia of all mice (n = 7 mice/age/genotype) (Table 1).

### Static microCT analysis of cortical mid-diaphysis

The volume of interest (VOI) analyzed was 5% of the tibial length, centered at the mid-shaft of the tibia and extended 2.5% along the bone’s long axis in the proximal and distal directions (Fig. 1). A global threshold of 4626 HU (809.6 mg HA/ccm) was used to distinguish cortical bone from soft tissue and from water. The investigated cortical bone parameters included: principal moments of inertia (Imax, Imin), cortical bone area (Ct.Ar), total cross-sectional area inside the periosteal envelope (Tt.Ar), cortical area fraction (Ct.Ar/Tt.Ar), cortical thickness (Ct.Th), and cortical volumetric tissue mineral density (Ct.vTMD) as described^42^.

### Dynamic microCT analysis of cortical mid-diaphysis

3D dynamic time lapsed *in vivo* morphometry was performed for all mice, whereby microCT images taken at day 0 and 15 are geometrically aligned and analyzed using a registration, segmentation and quantification algorithm (Fig. 1). The method has previously been described in detail^9, 43^. Briefly, the algorithm involves the following steps: 1) geometrical registration of images, 2) thresholding to extract the bone region, using the same global threshold mentioned above, 3) segmentation to exclude mineralized tissue present in the medullary cavity inside the VOI, 4) labeling regions of quiescent, newly formed and resorbed bone, and 5) quantification of volumetric dynamic (re)modeling parameters of formation and resorption normalized to values at the beginning of experiment (bone volume newly mineralized between day 0 and 15 divided by the bone volume present at day 0: MV/BV_day0–15_; bone volume eroded between day 0 and 15 divided by the bone volume present at day 0: EV/BV_day0–15_; mineralizing and eroded surface between day 0 and 15 normalized to the total bone surface at day 0: MS/BS_day0–15_ and ES/BS_day0–15_).

### Dynamic histomorphometry

Calcein (20 mg/kg) was administered to the LC mice via intraperitoneal injection at day 3 and 12. *Sost* KO mice were labeled with calcein at day 3 (20 mg/kg) and alizarin (30 mg/kg) at day 12. Although the labeling dyes were different between the genotypes, Sun *et al*.^44^ showed that calcein and alizarin staining provide comparable results. After dissection of the tibiae from the surrounding soft tissues, their lengths were measured using digital calipers to be the following: LC young: 17.3 ± 0.4 mm, LC adult: 18.4 ± 0.3 mm *Sost* KO young: 17.7 ± 0.3 mm and *Sost* KO adult: 18.4 ± 0.3 mm.

The tibiae were dehydrated in ascending grades of ethanol to absolute, cleared in xylene, infiltrated and finally embedded in methyl-methacrylate. The blocks were sectioned transversal to the bones long axis at the cortical midshaft. The slices were ground and polished to an approximate thickness of 60 μm and viewed at a magnification of 200 × under a mercury lamp microscope (KS400 3.0, Zeiss, Oberkochen, Germany) for evidence of fluorochrome labels (Figs 1, 5). Images were acquired using commercially available software (Axiovision, Zeiss, Oberkochen, Germany). The analyzed region of interest for the cortical bone included endo- and periosteal surface. The single- and double-labeled surface per bone surface (sLS/BS, dLS/BS), mineralizing surface (MS/BS), mineral apposition rate (MAR), and bone-formation rate (BFR/BS), were analyzed using ImageJ. MS/BS was calculated as 0.5x sLS/BS + dLS/BS. When a specimen had no double-labeled surface (dLS/BS = 0), it was labeled as “no data” for MAR and BFR/BS^45^. The amount of newly mineralized bone per day was calculated using the averaged double label distances divided by the 9-day labeling interval, and expressed as the MAR in units of microns per day. For determining MAR, the entire endocortical (Ec) and periosteal (Ps) surfaces were analyzed (Fig. 5). Except for the young *Sost* KO control limb (n = 6), 7 specimen per group were analyzed (Table 2).

### qPCR analysis

The left tibiae of an additional 72 female mice (n = 6/genotype/age/time point) was loaded for a single loading session. The loading procedure has been described above (see section *In vivo mechanical loading)*. Mice were sacrificed at either 3, 8, or 24 hours after the single loading session. Bone marrow was removed and RNA was extracted using TRIzol® reagent (Thermo-Fisher), followed by purification with RNEasy kit (Qiagen). RNA quality and concentration were verified by Nanodrop and reverse transcription was performed using iScript™ cDNA Synthesis Kit (Biorad). Gene expression was determined using a QuantStudio™ 7 Flex system, TaqMan® Fast Advanced Master Mix and the following TaqMan® probes (Thermo-Fisher): *Lef1* (Mm00550265_m1), *Axin2* (Mm00443610_m1), *Dkk1* (Mm00438422_m1), *Sost* (Mm00470479_m1), *Wnt1*(Mm01300555_g1), *Wnt7b* (Mm01301717_m1) and *B2m* (Mm00437762_m1) for normalization. Relative expression was calculated using the ΔCt method. For each condition, 5–6 animals were analyzed. It should be noted that we could not detect expression of Wnt ligands (*Wnt1* and *Wnt7b*).

### Statistical analysis for microCT parameters,histomorphometry and qPCR

The within-subject effect of loading (loaded, control limbs) and between-subject effects of age (10 week old, 26 week old) and genotype (*Sost* KO mice and LCs) as well as interactions between these terms was assessed using a repeated measures ANOVA (SAS 9.4, Cary, USA) for absolute values. A separate ANOVA was used to assess between-subject age (10 week old, 26 week old) and genotype (*Sost* KO mice and LCs) and interaction effects for relative values, the interlimb differences (∆_interlimb_ = loaded limb − control limb). Furthermore, subanalyses were performed on absolute values and relative values (interlimb difference) of all outcome measures using paired or independent t-tests. The percent difference is presented as %Δ = ((loaded limb – control limb)/control limb) × 100%). Statistical analyses of the qPCR analysis were performed on the ΔCt values (Fig. 6). A p value ≤0.05 was considered significant.
